# Teratogenic effect of *Lippia citriodora* leaves aqueous extract in mice

**Published:** 2016

**Authors:** Zahra Oskouei Shirvan, Leila Etemad, Reza Zafari, Seyed Adel Moallem, Naser Vahdati-Mashhadian, Hossein Hosseinzadeh

**Affiliations:** 1 *Departments of Pharmacodynamics and Toxicology, School of Pharmacy, Mashhad University of Medical Sciences, Mashhad, Iran*; 2 *Pharmaceutical Research Center, Mashhad University of Medical Sciences, Mashhad, Iran*; 3 *Medical Toxicology Research Center, School of Pharmacy, Mashhad University of Medical Sciences, Mashhad, Iran*; 4 *Pharmaceutical Research Center, Department of Pharmacodynamics and Toxicology, School of Pharmacy, Mashhad University of Medical Sciences, Mashhad, Iran*

## Abstract

**Objective:**

Safety of *Lippia citriodora*, as a herbal remedy, in pregnancy has not yet been evaluated. This study aimed to identify the effect of *L. citriodora* aqueous extract on pregnancy outcome in mice.

**Materials and Methods:**

Timed-pregnant mice received doses of 0.5 g/kg/day *L. citriodora* aqueous extract or the vehicle control during organogenesis, intraperitoneally. Maternal body weights were measured throughout the pregnancy. The litters were examined for external malformations and skeletal abnormalities. Fetuses were stained with Alizarin red S and Alcian blue.

**Results:**

There were no significant differences in mean maternal weight gain during pregnancy between groups. Also, no significant differences were observed in mean number of implantation, live and resorbed fetuses between control and treated groups. The prevalence of all types of deformity was low and similar to control group (%1.11).

**Conclusion:**

The results of this study show that moderate consumption of *L. citriodora* as an infusion or tea appears to be safe to be used during pregnancy and does not have toxic effects on development of mouse embryo.

## Introduction

The genus *Lippia* (Verbenaceae) includes approximately 200 species of herbs, shrubs and small trees (Quirantes-Pine et al., 2013 ▶). The plant lemon verbena (*Lippia citriodora and Aloysia triphylla*), a member of Verbenaceae family, is one of the most important species mainly used as a spice and herbal medicine (Valentao et al., 2002 ▶). It is a shrub indigenous to South America and is cultivated in northern Africa, southern Europe and north of Iran (Quirantes-Pine et al., 2013 ▶). It has been traditionally used in treatment of colds, fever, asthma, colic, diarrhea, indigestion, anxiety and insomnia. Due to the lemon aroma, lemon verbena leaves are traditionally utilized as infusions for their digestive, antispasmodic, sedative and antipyretic properties (Pascual et al., 2001 ▶; Valentao et al., 2002 ▶). Indeed, the leaves of *L*. *citriodora* are used for flavoring beverages and culinary seasoning. Modern pharmacological studies have demonstrated that the leaves of this species have antispasmodic, digestive, sedative, antipyretic and stomachic properties (Valentao et al., 2002 ▶). 

Most pharmacological properties have been attributed to the essential oil rich in phenolic compounds, namely phenyl propanoids and glycosilated flavones (Moradi et al., 2014 ▶). Verbascoside, the most abundant phenyl propanoid glycoside from lemon verbena is reported to possess many biological features including anti-oxidative, anti-bacterial, anti-tumor and anti-fungal actions (Pastorelli et al., 2012 ▶).

Considering the widespread therapeutic and non-therapeutic application of lemon verbena; its consumption during pregnancy may be more likely to happen. But information on the safety of *L.citriodora* in pregnancy has not yet been evaluated in the scientific or traditional literatures (Gardner and McGuffin, 2013 ▶). Therefore, this study was done to identify effect of *L. citriodora* aqueous extract on pregnancy outcome in mice.

## Materials and Methods

**Animal**

Female BALB/c Mice, 10-12 weeks of age obtained from animal center, School of Pharmacy, Mashhad University of Medical Sciences, were maintained in an environmentally controlled room (18- 22 °C) with a 12-h light/12-h dark cycle. Laboratory mouse chow and tap water were provided *ad libitum*.

**Plant material**

*L. citriodora* leaves were collected from the surrounding areas of Karaj city, Alborz province, Iran, dried in shadow and grinded to make powder. *L. citriodora* was properly identified by the Department of Botany in Ferdowsi University, Mashhad, Iran and voucher samples were preserved for reference in the herbarium of the Department of Pharmacognosy, School of Pharmacy, Mashhad University of Medical Sciences, Mashhad, Iran.

**Preparation of aqueous extract**

Aqueous extract of *L. citriodora* was prepared by adding 1L of distilled water to 100 g of powdered plant material in a 2.5L glass flask and boiled for 20 min. The solution was subsequently filtered using Whatman No. 1 filter paper and then concentrated under vacuum at 46 °C using a rotary evaporator. The residues obtained were stored in a freezer at −55 °C until use. 

**Animal treatment**

Two female and one mail mice of the same strain were caged for one night and the day of vaginal plug observation was considered as day 0 of gestation (GD0).

Treated and control pregnant mice, 10 in each group, received a single intra peritoneal (IP) injection of 0.5 g/kg body weight *L. citriodora* aqueous extract dissolved in normal saline and the equivalent volume of solvent by the same route, during GD6–15 (organogenesis period), respectively. For dose selection, median lethal dose and maximum tolerated doses (MTD) of extract were determined and 30% of MTD was chosen as the administered dose.

**Maternal and fetus observation**

During the experiment, all groups were evaluated daily for mortality, morbidity and general appearance. Maternal body weights were measured throughout the pregnancy. Maternal body weight gain was calculated by subtracting the weight of pregnant mice on GD0 from that on GD18. On the 18th day of pregnancy, cesarean section was performed under anesthesia. The embryos were carefully removed from uterine and examined under dissecting stereomicroscope for external malformations including abdominal hernia, polydactyl, anencephaly, cleft palate, etc. The size (crown-rump length) and body weight of fetuses were also measured. The detection of skeletal anomalies was performed by whole mount skeletal staining with Alizarin red S and Alcian blue using Kimmel and Trammell methods (Kimmel and Trammell, 1981 ▶).

**Statistical analysis**

The body weight and crown-rump length were presented as mean ± SEM. T-test was performed to make comparisons between control and experimental group. Differences between frequencies of the absorbed and live fetuses and external malformation between the groups were compared using Fisher’s direct probability test and when the frequency of each category was 5 or more, the Chi-Square test was used. Data were analyzed using SPSS software (version 11.5).

## Results

**Maternal observation**

**Figure 1:**
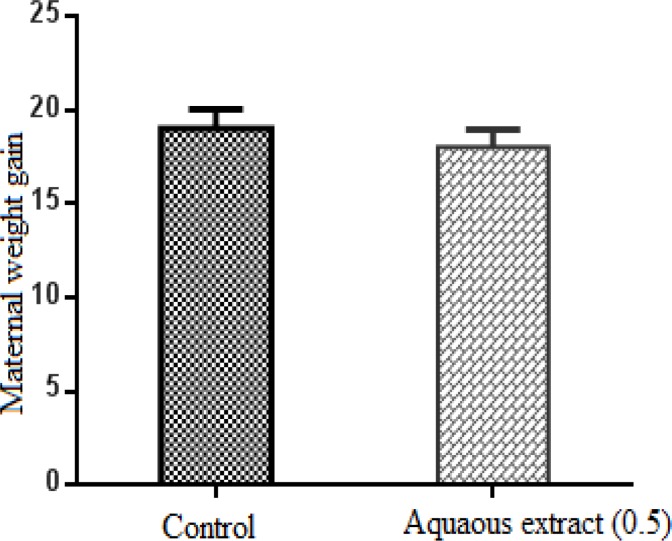
Comparison between maternal weight gains during pregnancy in control and treated groups that received normal saline and 0.5 g/kg *L. citriodora *aqueous extract, respectively. Data are shown as mean ± SEM. The difference between two groups is not statistically significant (T-test).

Pregnant mice were all alive at the time of the cesarean section. The mean maternal weight gain during pregnancy was 19.04 ± 1.01 and 18.03 ± 0.94 g in control and treated groups, respectively. *L. citriodora* aqueous extract administration during pregnancy resulted in no significant changes in body weight (Figure 1). Behavior signs and water or food consumption in dams of both groups also did not change. No significant differences were observed in the mean number of implantation, live and resorbed fetuses between control and another group (Table1).

**Fetus observation**

**Figure 2:**
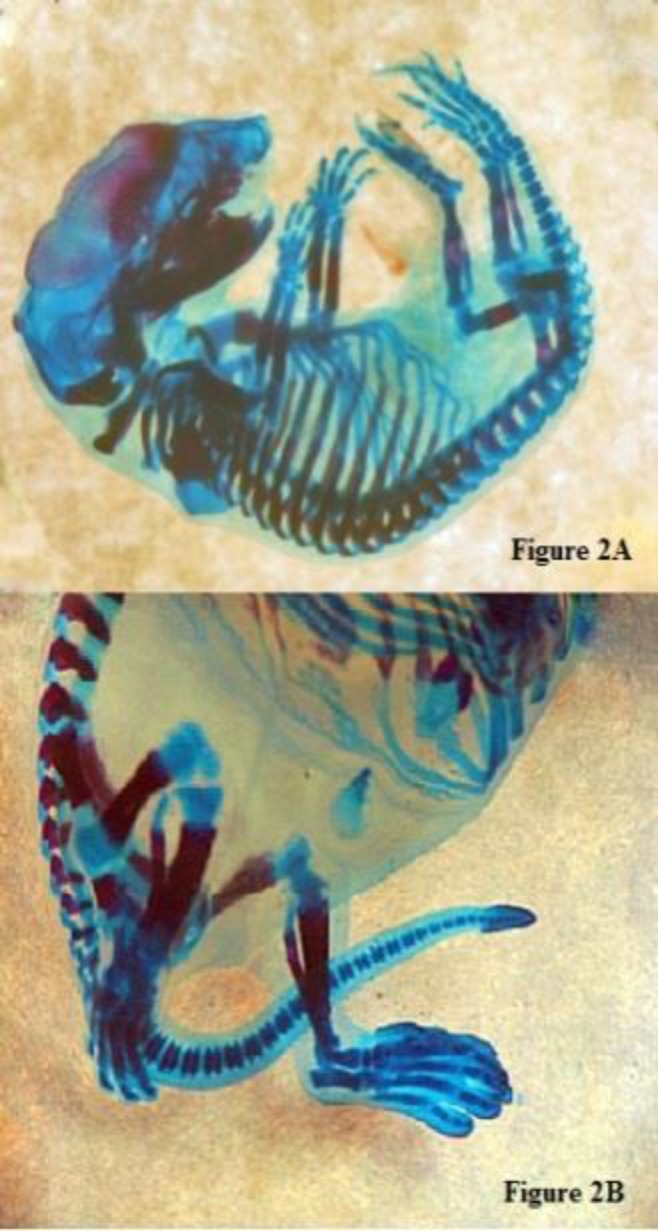
Fetus after skeletal staining (A) with scoliosis and limb deformity (B) in the group treated with 0.5 g/kg *L. citriodora* aqueous extract intra peritoneal injection, which was stained with Alizarin red S-Alcian blue

Exposure to lemon verbena at a dose of 0.5 g/kg/day also had no effect on the mean fetal weight and mean crown-rump length (Table 1). The incidence rates and types of malformations detected in fetuses treated with aqueous extract of *L. citriodora* are shown in Tables 2. The abnormalities included vertebral column deformity, limb deformity and craniofacial abnormality (Figure 2). The prevalence of all types of deformity was low and similar to extract-treated group (%1.11). The total number of defects in treated group compared to control group was higher, although the difference was not significant.

**Table 1:** Cesarean section parameters in BALB/c mice fetuses exposed to *L. citriodora* aqueous extract

**Treatment and dose (mg/kg/day)**		
	Control (Normal saline)	Treated group (0.5 g/kg)
**Dams, No**	10	10
**Number of implantation, Mean ± SEM**	118 (11± 0.61)	95 (9.3± 0.5)
**Live fetuses, No (%)**	115(97.45)	90(94.73)
**Resorbed fetuses, No (%)**	3 (2.54)	5 (5.26)
**Fetal weight, Mean ± SEM (g)**	1.08± 0.03	1.14±0.02
**Fetal length, Mean ± SEM (mm)**	18.58±0.42	19.42±0.26

Data are shown as mean ± SEM or percentage. The difference between the two groups is not statistically significant (T-test or Fisher's Exact Test).

Figure1. Comparison between maternal weight gains during pregnancy in control and treated groups that received normal saline and 0.5 g/kg *L. citriodora* aqueous extract, respectively. Data are shown as mean ± SEM. The difference between two groups is not statistically significant (T-test).

**Table2:** Skeletal malformations in BALB/c mice fetuses exposed to *L. citriodora* aqueous extract

**Treatment and dose (mg/kg/day)**		
	Control (Normal saline)	Treated group (0.5 g/kg)
**Dams, No**	10	10
**Fetuses examined, No**	115	90
**Vertebral column deformity, No (%)**	0	1 (1.11)
**Limb deformity, No (%)**	0	1 (1.11)
**Craniofacial abnormalities, No (%)**	0	1 (1.11)
**Total number of birth defect, No (%)**	0	3 (3.33)

Data are shown as mean ± SEM or percentage. The difference between the two groups is not statistically significant (Fisher's Exact Test

## Discussion

World Health Organization reports that up to 80% of the population uses herbal remedies (Ansari et al., 2012 ▶). *L. citriodora*, a plant native to South America, has been widely used in folk medicine and also for industrial purposes. Multiple medicinal uses have been suggested for this plant. Some of its pharmacological activities including analgesic, anti-inflammatory, antipyretic and sedative have been confirmed (Pascual et al., 2001 ▶). Nevertheless, there is not yet enough information available to determine the side effects and risk of *L.citriodora* in pregnancy. The result of our study revealed that administration of the aqueous extract of *L.citriodora* during the period of organogenesis has not been associated with fetal malformations, and no signs of maternal toxicity were observed. However, the teratogenic effects of some plants in the Verbenaceae family have been confirmed. For instance, administration of *Lantana camara* hydroalcoholic extract to pregnant rats induced skeleton anomalies by post-implantation loss (Mello et al., 2005 ▶). 

Scientific explorations have shown that some of herbal remedies may have toxic effects on fetus development. Research on a widely used herb, Saffron has revealed that crocin and safranal (two main components of this plant) can induce embryonic abnormalities particularly skeletal malformations when administered to pregnant mice (Moallem et al., 2013 ▶). Also, it has been suggested that *Salvia leriifolia* Benth consumption during pregnancy may cause some abnormalities such as spina bifida, limb abnormalities, abdominal bleeding and bone abnormalities (Hosseinzadeh et al., 2009 ▶).

International Fragrance Association (IFRA) states that the essential oil from the fresh herb extract might sensitize the skin to sunlight (phototoxicity)(Dweck, 2009 ▶). 

Lemon verbena has been used in traditional medicine to treat menstrual cramps (primary dysmenorrhea) in Mexico. This effect was attributed to antispasmodic effect of *L. citriodora* hexane extract. The hexane extract relaxed the uterus by inhibiting the contractile response induced by PGF_2_ (Ponce-Monter et al., 2010 ▶)_._ One of the most important mechanisms that has been postulated to explain the cause of teratogen-induced fetal malformations is excessive formation of reactive oxygen species or impaired antioxidant defense (Wells et al., 2005 ▶). It has been shown that supplementation of antioxidants can attenuate teratogen-induced oxidative stress and thus reduce fetal damage. There is substantial evidence to support that oxidative stress is one of the most important mechanism by which fetal alcohol syndrome occurs. The research result showed that treating alcoholic women with antioxidants such as vitamins C and E may potentially prevent ethanol teratogenicity (Cohen-Kerem and Koren, 2003 ▶). Also, it is indicated that some herbs, like ginger, with high antioxidant activity has no teratogenic effects (Masuda et al., 2004 ▶; Fischer-Rasmussen et al., 1991 ▶). Therefore, the high antioxidant activity that was attributed to *L. citriodora* may partly explain our results (Valentao et al., 2002 ▶). 

In conclusion, this study showed that moderate consumption of *L. citriodora* as an infusion or tea appears to be safe during pregnancy and does not have toxic effects on development of mouse embryo. However, additional research is needed to determine the exact effects of *L. citriodora* on human embryo development.
